# Enigma of Extrapulmonary Tuberculosis: Where Do We Stand?

**DOI:** 10.7759/cureus.1554

**Published:** 2017-08-09

**Authors:** Faisal Inayat, Munnam S Jafar, Nouman Safdar Ali, Qulsoom Hussain, Abu Hurairah

**Affiliations:** 1 Department of Medicine, New York-Presbyterian Hospital, Weill Cornell Medical College, New York City, NY, USA; 2 Department of Medicine, Jinnah Hospital, Allama Iqbal Medical College, Lahore, Pakistan; 3 Department of Medicine, Shifa International Hospital, Shifa College of Medicine, Islamabad, Pakistan; 4 Division of Gastroenterology, Department of Medicine, SUNY Downstate Medical Center, Brooklyn, New York

**Keywords:** extrapulmonary tuberculosis, diagnosis, treatment, lymphadenopathy, abdominal involvement, awareness, public health

## Abstract

Tuberculosis remains a worldwide public health concern. Atypical extrapulmonary presentations may delay its diagnosis and treatment. The present study illustrates the importance of ruling out extrapulmonary tuberculosis in patients presenting with nonspecific symptoms of abdominal diseases. Furthermore, we discuss the variety of clinical presentations, diagnostic challenges, current therapeutic protocols, and prognostic factors associated with extrapulmonary tuberculosis. Early diagnosis and effective treatment may decrease morbidity and mortality in such patients.

## Introduction

Tuberculosis (TB) can involve any organ, with lungs being the most common. Extrapulmonary tuberculosis (EPTB) is the term used to describe the occurrence of TB at body sites other than the lungs [1]. It commonly involves lymph nodes (19%), pleura (7%), gastrointestinal tract (4%), bone (6%), central nervous system (3%), and genitourinary system (1%) [2]. The total TB cases in industrialized countries are constantly declining. However, the reduction in EPTB is relatively smaller [3].

Mycobacterium tuberculosis(MTB) spreads through airborne particles, called droplet nuclei, 1-5 microns in diameter, from close contact with the active disease. After an effective immune response in the majority of individuals, the bacteria become dormant, resulting in latent tuberculosis infection (LTBI). These infected individuals are asymptomatic and not infective. One-third of the world's population has LTBI, which can progress to an active state [4]. Recognized risk factors for developing TB include proximity to an infectious case and bacillary load, immunosuppressive conditions, such as human immunodeficiency virus (HIV) infection, diabetes, smoking, alcohol, young age, malnutrition, and indoor air pollution [5].

## Case presentation

A 53-year-old female with a history of hypertension and diabetes mellitus presented to our medical center with epigastric pain and intermittent fever for two months. The pain was moderate in intensity and was associated with loss of appetite, night sweats, and a 20-pound unintentional weight loss. There was no history of nausea, vomiting, shortness of breath, cough, or change in bowel habits, and she did not undergo any surgical procedures. She was a nonsmoker, nonalcoholic, and drug-free. The patient immigrated to New York City from Haiti five years ago.

On physical examination, she had tachycardia with heart rate at 108 beats per minute and fever of 103.7°F. There were no signs of acute distress and she was alert and oriented. Her chest was clear to auscultation bilaterally. The abdomen was soft and nondistended with mild epigastric tenderness. Bowel sounds were normal in pitch and frequency. A complete blood count revealed normal levels, except for a white cell count of 3,790 cells/mcL (normal, 3,500 to 10,500 cells/mcL). Electrolytes and liver function tests were within normal limits. Serum amylase and lipase levels were also normal. HIV testing came back negative. The purified protein derivative (PPD) skin test was positive with an induration of 15 millimeters.

**Figure 1:**
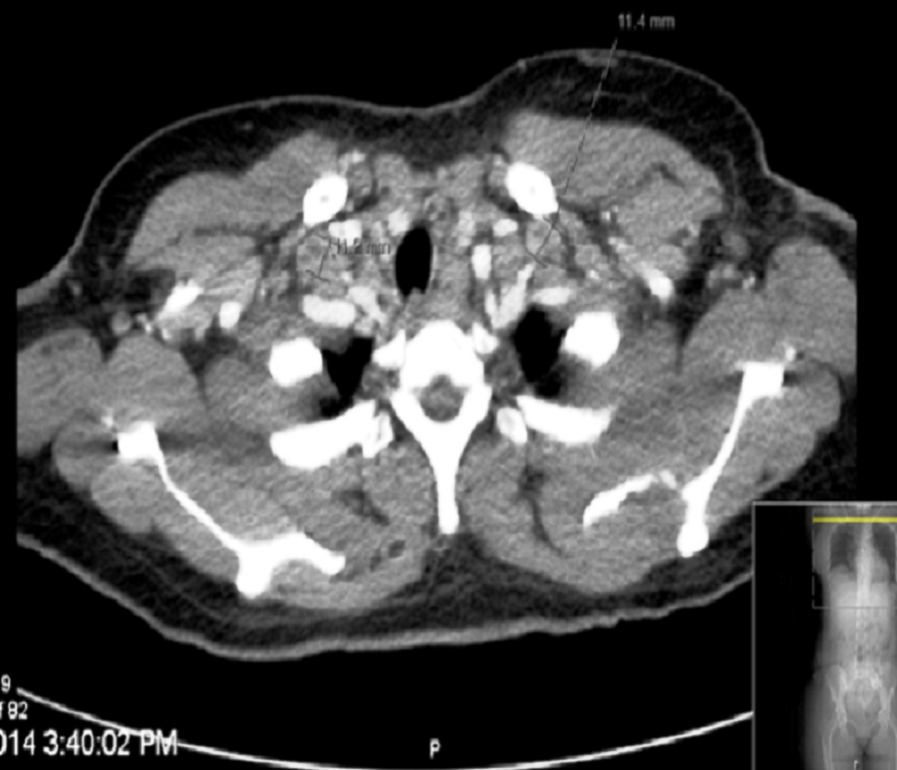
CT chest CT chest revealing right paratracheal, prevascular, subcarinal, and bilateral supraclavicular lymphadenopathy

Subsequently, the patient underwent a series of radiological investigations. Her chest radiograph had no abnormal findings. A high resolution computed tomography (CT) scan of the chest showed lymphadenopathy involving the right paratracheal, prevascular, subcarinal, and bilateral supraclavicular nodes with no lung consolidation (Figure 1). 

**Figure 2:**
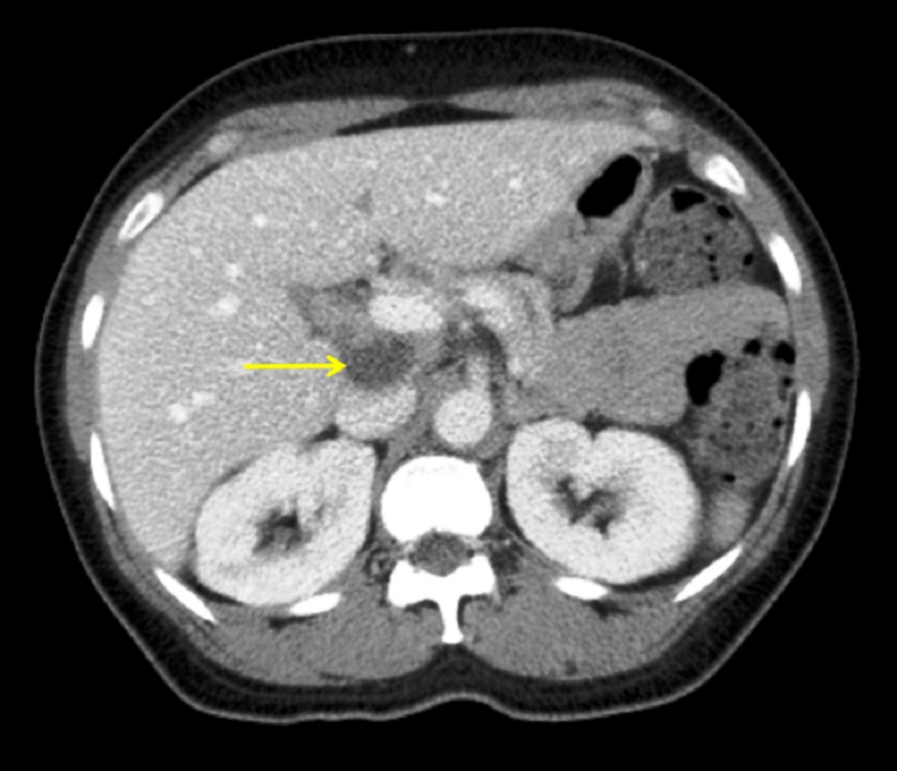
CT abdomen (transverse section) CT abdomen showing retrocardiac lymphadenopathy

Abdominal CT scan revealed retrocardiac lymphadenopathy (Figure 2).

**Figure 3:**
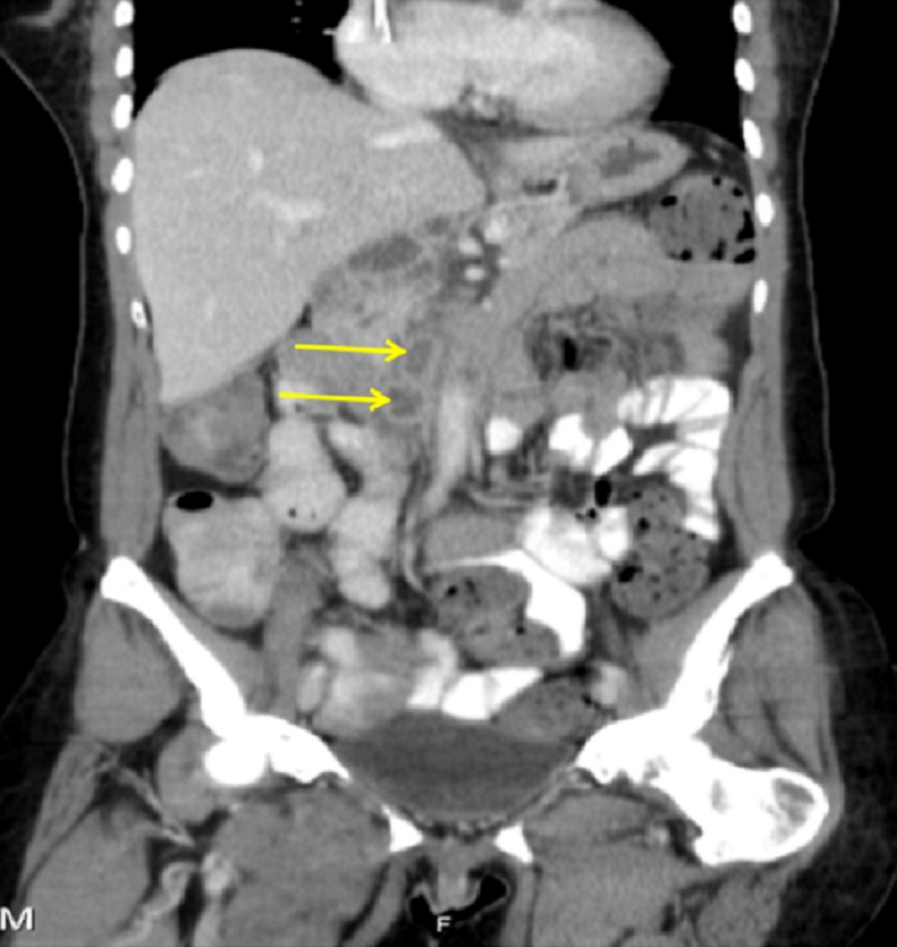
CT abdomen and pelvis (transverse section) CT abdomen and pelvis showing left para-aortic and aortocaval lymph node enlargement, measuring 1.2 and 1.4 centimeters in diameter, respectively. Mesenteric or pelvic lymphadenopathy was not present.

On CT abdomen and pelvis, the left para-aortic and aortocaval lymph nodes appeared enlarged, measuring 1.2 and 1.4 centimeters in diameter, respectively. There were no abnormally enlarged mesenteric or pelvic lymph nodes (Figure 3). 

**Figure 4:**
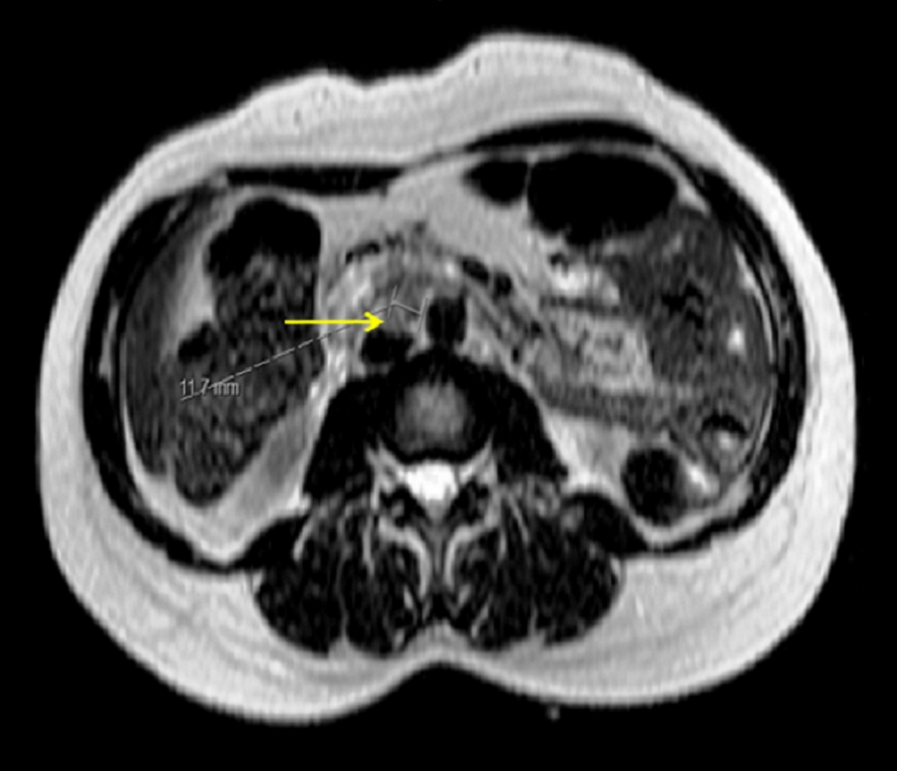
MRI abdomen On MRI abdomen, retroperitoneal and para-aortic lymph nodes appeared enlarged up to 1.3 centimeters. A 1.2-cm para-aortic lymph node was also seen just inferior to the duodenum. No mesenteric lymphadenopathy was present and there was no focal lesion in the pancreas or liver.

Magnetic resonance imaging (MRI) abdomen revealed retroperitoneal and para-aortic lymph nodes enlargement up to 1.3 centimeters in diameter. A para-aortic lymph node was noted just inferior to the duodenum, measuring 1.2 centimeters in diameter. There was no evidence of mesenteric lymphadenopathy and no focal lesion was identified in the pancreas or liver (Figure 4).  

**Figure 5:**
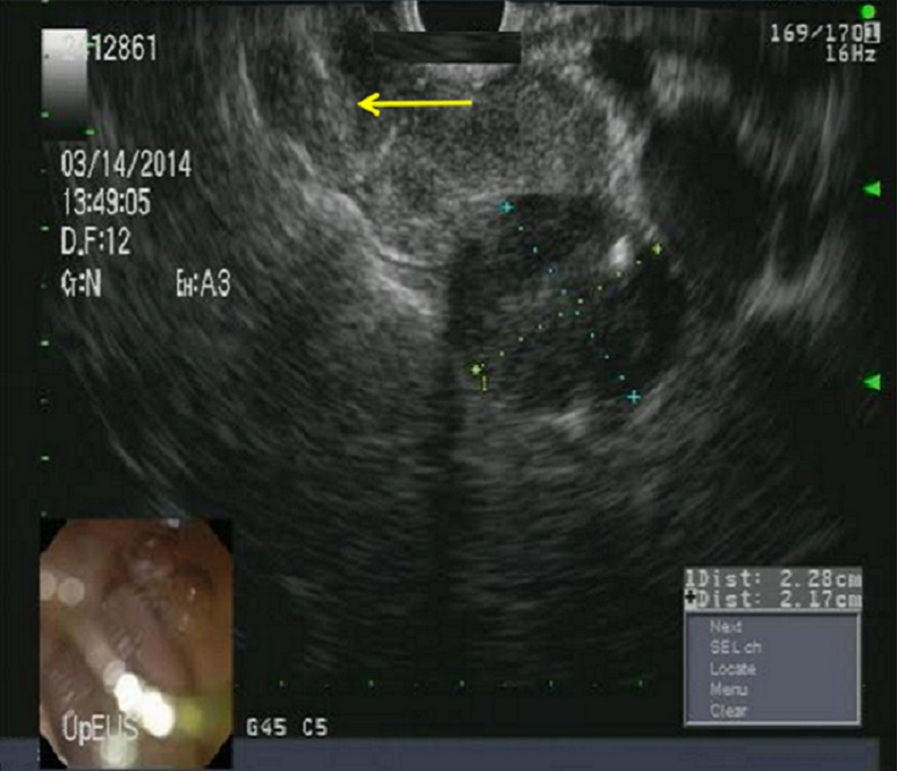
Endoscopic ultrasound Multiple enlarged peri-hepatic and peri-portal lymph nodes were identified on endoscopic ultrasound and a peri-hepatic lymph node was aspirated and biopsied (demarcated by the yellow arrow).

Sputum acid-fast bacilli (AFB) staining and microscopic examination came back negative three times. Therein, a peri-hepatic lymph node measuring 2.3 x 1.7 centimeters in size was located using endoscopic ultrasonography (Figure 5). 

**Figure 6:**
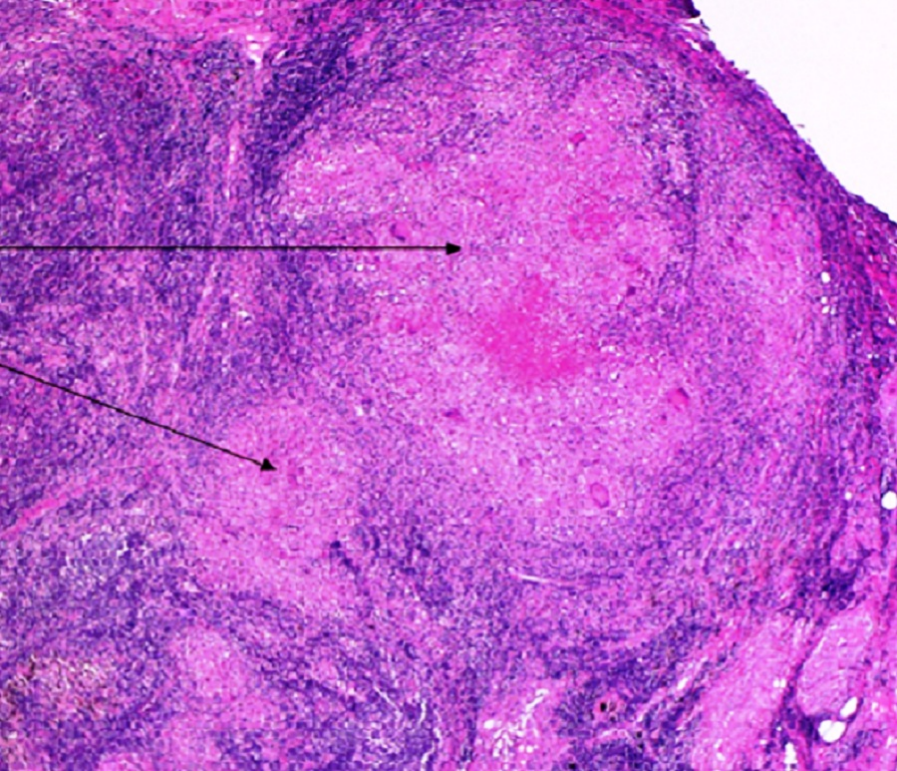
Histopathology On histopathologic analysis, tuberculous lymphadenitis with extensive necrosis was noted. Granuloma formation was identified (marked by black arrows). Acid-fast staining showed acid-fast bacilli.

It was aspirated and biopsied using a 22G fine-needle aspiration (FNA) needle (Boston Scientific, Natick, MA) and a 22G Procore needle (Boston Scientific, Natick, MA), respectively. Samples were sent for cytological, histopathologic, and microbiological analysis. Analysis of the FNA sample showed tuberculous lymphadenitis with necrosis and there was evidence of granuloma formation. Rare bacilli were noted on acid-fast staining (Figure 6). 

**Figure 7:**
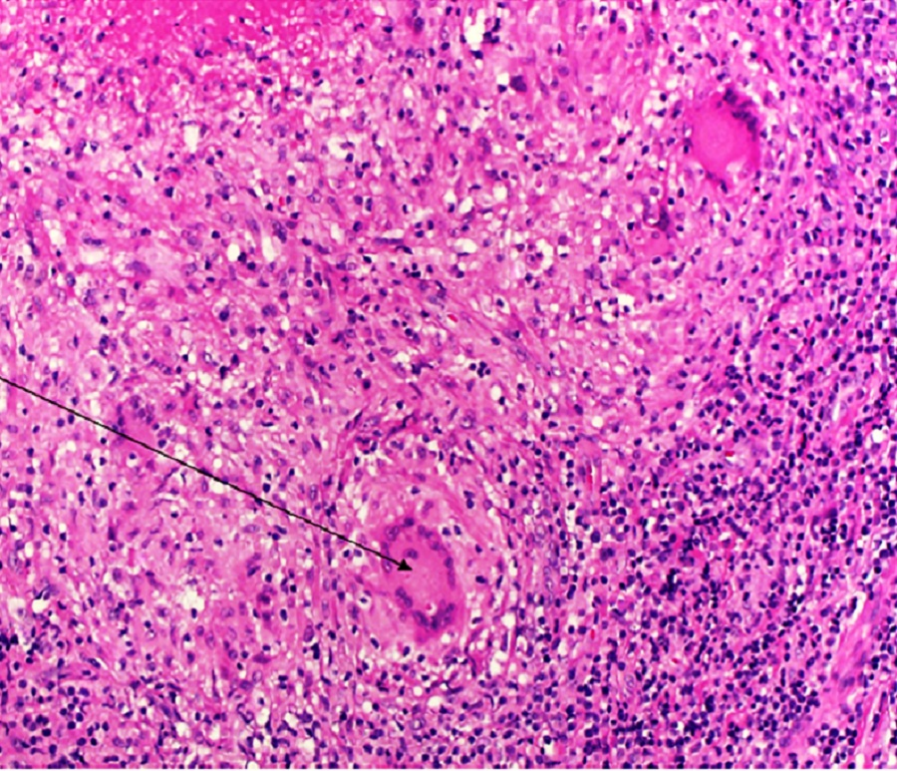
Histopathology Histopathologic examination of the biopsy specimen showed necrotizing tuberculous lymphadenitis with a few giant cells (marked by black arrow). TB lymphadenitis with necrosis was present and rare bacilli were seen upon acid-fast staining.

Analysis of the biopsy specimen revealed necrotizing tuberculous lymphadenitis with extensive necrosis. AFB staining of the biopsy sample revealed acid-fast bacilli (Figure 7).

The patient was discharged from the hospital on isoniazid, rifampin, ethambutol, pyrazinamide, and vitamin B6 and continued to improve with close outpatient follow-up. After six months of treatment, the patient recovered completely and continues to do well.

## Discussion

Robert Koch's (1843-1910) statement that TB is a disease deadlier than the plague or cholera holds true even today [1]. In the year 2013, nine-million people were infected and 1.5-million deaths occurred owing to TB [2-3]. It is a disease without boundaries and can present anywhere, but is associated with a higher mortality in low- and middle-income, resource-limited countries [4]. Lymph node tuberculosis (LNTB) is the most prevalent form of EPTB in developing countries, whereas non-tuberculous mycobacteria (NTB) constitute the most common cause of lymphadenopathy in the developed world. LNTB most frequently involves the cervical lymph nodes followed by mediastinal, axillary, mesenteric, hepatic portal, perihepatic, and inguinal lymph nodes [5]. It presents with symptoms that vary with the anatomical site involved: dysphagia and recurrent laryngeal nerve palsy may be the presenting complaints in mediastinal TB, whereas periportal, mesenteric, or peripancreatic node involvement often manifests with nonspecific abdominal pain [2,5].

The usual presentations of abdominal TB include abdominal pain (80%-95%), weight loss (40%-90%), fever (40%-70%), diarrhea (11%-20%), constipation, alternating diarrhea and constipation, anorexia, and malaise [6]. The character of the pain is constant-dull in mesenteric LNTB and colicky in luminal compromise. In rare cases, abdominal TB may also present with a mid-esophageal ulcer, dysphagia, and odynophagia in esophageal TB; dyspepsia and gastric outlet obstruction in gastroduodenal TB; lower abdominal pain, and haematochezia in colonic TB [7]. The present case is unique, as the chief presenting complaint in our patient was epigastric pain that has a broad differential diagnosis and is rarely attributed to lymphadenopathy. To our research, our patient represents the first case of EPTB with an initial presentation related to epigastric pain.

Owing to the nonspecific nature of the presenting symptoms and inconsistent physical and laboratory findings, the diagnosis of LNTB is a challenge. Tuberculin skin test (TST) and interferon gamma release assay (IGRA) are used to screen for LTBI. IGRA has higher specificity than TST; however, both of these tests lack the ability to distinguish between LTBI and an active disease [5, 8]. Abdominal lymphadenopathy, the most common manifestation of abdominal TB, is best evaluated on CT. It reveals enlarged lymph nodes with hypoattenuating centers and hyperattenuating enhancing rims [8]. Furthermore, chest radiography, ultrasonography, and MRI can demonstrate lymphadenopathy and may help in planning a diagnostic intervention. A cytological exam of FNA samples of lymph nodes carries a sensitivity and specificity of 88% and 96% for the diagnosis of tuberculous lymphadenitis, respectively [9]. As EPTB is a paucibacillary disease, the sensitivity is improved by polymerase chain reaction (PCR) testing, which can detect as few as 10 mycobacteria [1, 9]. PCR employed on samples obtained by FNA or biopsy has a 43%-84% sensitivity and a 75%-100% specificity, obviating the need for an open biopsy. It is particularly helpful in smear and culture-negative cases [9].

Xpert® MTB/resistance to rifampin (MTB/RIF) (Cepheid, Sunnyvale, CA) is a rapid DNA test that detects the presence of the DNA of MTB and mutations in the rpoB gene in ~2 hours. This not only helps in identifying rifampicin resistance by detecting rpoB mutations but also carries an overall sensitivity of 77.3%-95% and a specificity of 99%-100% in extrapulmonary samples (which is equivalent to TB liquid cultures) [10]. Xpert is highly sensitive for TB detection in lymph node samples with a pooled sensitivity of 83.1% (95% confidence interval (CI), 71.4–90.7%) *versus* culture and 81.2% (95% CI 72.4–87.7%) *versus *composite reference standard (CRS). It was moderately sensitive for the detection of TB meningitis (pooled sensitivity 80.5% (95% CI 59.0–92.2%) against culture and 62.8% (95% CI 47.7–75.8%) against CRS, but has lower sensitivity for testing pleural fluid (pooled sensitivity 46.4% (95% CI 26.3–67.8%) against culture and 21.4% (95% CI 8.8–33.9%) against CRS. Owing to these results, Xpert is recommended by the World Health Organization over conventional tests for the diagnosis of TB in lymph nodes and other tissues, and as the preferred initial test for the diagnosis of TB meningitis [10]. Histopathology and mycobacterial culture are diagnostic. However, it takes several weeks to obtain a growth on the culture, which may prolong the initiation of treatment.

The MTB has a high spontaneous mutation rate. Therefore, the treatment of an active infection requires a complex regimen consisting of multiple drugs. EPTB (except for meningeal, pericardial, and bone and joint disease) is treated with a six-month course, consisting of isoniazid (H), rifampin (R), pyrazinamide (Z), and ethambutol (E) with a combination of HRZE for the first two months followed by four months with HR [1-6]. The challenge of EPTB does not end with diagnosis and the initiation of treatment, as no defined guidelines exist to evaluate the treatment response. Residual lymph nodes have been used for assessing the treatment outcomes for LNTB. However, these are not reliable because their size can decrease more after the completion of the therapeutic regimen and 11%–13% patients are still left with long-term residual nodes [1]. ^18^F-fluorodeoxyglucose (FDG) PET/CT imaging is a promising modality, which may help in overcoming this challenge. 18F-FDG tends to accumulate in inflammatory cells in tuberculous lesions. The changes in ^18^F-FDG uptake, which correlate with clinical markers of response to therapy, can be used to predict the treatment response with high sensitivity and specificity, especially in patients with EPTB [7-10].

## Conclusions

The present paper highlights that EPTB is a great masquerader. It can present with a broad range of initial manifestations, varying according to the body site involved; therefore, its diagnosis requires a high index of clinical suspicion. EPTB should be included in the differentials in patients presenting with constitutional symptoms, especially in those hailing from endemic areas. Furthermore, the current diagnostic modalities enable us to establish an accurate diagnosis in suspected cases of EPTB and treatment response can be monitored with good precision in diagnosed cases. 

## Appendices

The patient agreed to participate and was explained the nature and objectives of this study, and informed consent was formally obtained. No reference to the patient's identity was made at any stage during data analysis or in the report.
